# Supplementary material for: Updated-Food Choice Questionnaire: Cultural Adaptation and Validation in a Spanish-Speaking Population from Mexico
Source: Nutrients. 2024 Oct 31;16(21):3749. doi: 10.3390/nu16213749 (PMC11548158; doi:10.3390/nu16213749)
Supplement: Supplementary file 1 [file nutrients-16-03749-s001.zip › Supplement S1. Face and content validity description.pdf]

Supplement S1. Material and methods for face and content validation

a) Face validation phase

In this phase, a panel of NP from the Health Sciences Center (Centro Universitario de Ciencias de la Salud – CUCS) from the Guadalajara University (*Universidad de Guadalajara*), in Mexico, was formed. Participants were required to meet the following inclusion criteria: a bachelor's or master's degree, clinical practice and nutritional research experience, not being participants of the study, and to voluntary agreeing to sign the informed consent form.

The NP received an e-mail during the period from April to June 2021 with an explanation of the purpose of the study, instructions for their participation, the informed consent, and a link to answer an online form containing the translated questionnaire. Each item was assessed dichotomously (yes/no) according to the four following criteria, proposed by Galicia-Alarcón et al. [1]: 1) if the item was clear (the item is easily understood, and its syntax and semantics are adequate); 2) relevant (the item is essential or important, and must be included); 3) specific (the item has its own character and its especially suitable for what it is intended to evaluate); and, 4) representative (the item can be considered characteristic of the aspect to be assessed).

Additionally, NP had the possibility to include open comments or suggestions if they consider it necessary. Afterwards, the research team developed a document containing the participants' suggestions received in this phase. Based on these comments and observations, the research team made adaptations to the wording of the items to make them more suitable for the Mexican population.

b) Content validation phase

Participants were considered from a group of academics who are regularly consulted for various topics by the Coordination of the Nutrition Bachelor's degree program from the University. Additionally, members of the first panel were also invited to participate.

The objective of this panel was to evaluate whether each one of all the items (original and new ones) met the aforementioned criteria (clarity, representativeness, relevance and specificity); however, in this phase, the four elements were grouped into a single question, with a dichotomous response (yes/no) and a space to specify which of the criteria was not fulfilled, including an area for comments or suggestions. A similar online form was used for the answering process. If the majority of the NP considered the item to be unclear, its wording was modified, whereas if the item was considered not relevant/specific/representative, the authors discussed whether the item was kept or eliminated. In addition, if the participants considered it necessary to add a new item, the authors deliberated whether or not it was included, according to the literature review and to our critical thinking.

References

1. Galicia-Alarcón, L.; Balderrama-Trapaga, J.; Navarro, R. Validez de Contenido Por Juicio de Expertos: Propuesta de Una Herramienta Virtual. *Apertura* **2017**, *9*, 42–53, doi:10.18381/ap.v9n2.993.
