# Supplementary material for: Updated-Food Choice Questionnaire: Cultural Adaptation and Validation in a Spanish-Speaking Population from Mexico
Source: Nutrients. 2024 Oct 31;16(21):3749. doi: 10.3390/nu16213749 (PMC11548158; doi:10.3390/nu16213749)
Supplement: Supplementary file 1 [file nutrients-16-03749-s001.zip › Supplement S2. Updated-Food Choice Questionnaire in English & Spanish (erratum).pdf]

Supplement S2-1. Updated-Food Choice Questionnaire (U-FCQ) in English.

Please select for each item only the option that represents, to the greatest extent, the degree to which you agree with the corresponding statement, "It is important to me that the food I eat on a typical day..." Using the following options: "Not at all important"; "Not very important"; " A little important"; "Moderately important"; or "Very important".

**Item**	**Importance**			
All items begin with the following statement: *"It is important to me that the food I eat on a typical day..."*	Not at all	A little	Moderately	Very
1. Is gluten-free.				
2. Is part of the traditional Mexican diet.				
3. Is produced by companies I recognize from television, social media or others.				
4. Is low in sugar.				
5. Tastes good.				
6. Has the shortest storage time (as fresh as possible).				
7. Are local and regional products.				
8. Is considered good by other people.				
9. Keeps me full for a considerable period of time.				
10. Has not been transported long distances.				
11. Can be bought close to where I live or work.				
12. Is not expensive.				
13. Is rich in fiber.				

Item	Importance			
All items begin with the following statement: *"It is important to me that the food I eat on a typical day..."*	Not at all	A little	Moderately	Very
14. Has eye-catching advertising on their packaging.				
15. Helps me to meet my energy and nutritional needs.				
16. Is in line with my religious beliefs.				
17. Is familiar or known to me.				
18. Is cheap.				
19. Is produced without animals having suffered pain.				
20. Is what I usually eat.				
21. Is free of non-caloric sweeteners, for example: sucralose, stevia, etc.				
22. Is recommended by health professionals.				
23. Is like the food I ate when I was a child.				
24. Is on sale.				
25. Smells nice.				
26. Is easy to digest.				
27. Are seasonal products (typical of the season of the year, such as certain fruits and vegetables).				
28. Is low in fat.				
29. Reduces soil and water products.				
30. Has a pleasant texture.				
31. Is rich in protein (e.g., eggs, beans, meat).				

**Item**	**Importance**			
All items begin with the following statement: *"It is important to me that the food I eat on a typical day..."*	Not at all	A little	Moderately	Very
32. Helps me get through life.				
33. Is low in animal fat.				
34. Keeps me awake/alert.				
35. Is consumed by the majority of the members of my household.				
36. Has the least number of seals of warning (i. e. excess sodium, calories, saturated fat).				
37. Can be easily cooked.				
38. Is produced respecting animal rights.				
39. Has an easy-to-understand nutrition labeling.				
40. Sticks to the “Plato del Bien Comer” guide (food guide from Mexico).				
41. Is low in salt.				
42. Can be consumed in the company of other people.				
43. Can be consumed almost immediately.				
44. Does not cause environmental damage.				
45. Looks nice.				
46. Is rich in vitamins and minerals.				
47. Can be cooked quickly.				
48. Does not cause me physical discomfort (allergies, stomach ache, diarrhea, etc.).				
49. Is packaged in an environmentally friendly way.				

**Item**	**Importance**			
All items begin with the following statement: *"It is important to me that the food I eat on a typical day..."*	Not at all	A little	Moderately	Very
50. Is not canned.				
51. Is organic (free of fertilizers and pesticides).				
52. Reflects a positive image of me.				
53. Is easily available in stores, supermarkets, "tianguis", etc.				
54. Helps me reduce stress.				
55. Contains no artificial ingredients (preservatives, colorings, food substitutes/imitations, etc.).				
56. Is nutritious.				
57. Contains mostly natural ingredients.				
58. Is good for my skin/teeth/hair/nails, etc.				
59. Is pleasant to me.				
60. Helps me to manage my weight.				
61. Is homemade.				
62. Cheers me up.				
63. Is minimally processed.				
64. Has the country of origin clearly marked.				
65. Is low in calories (allows me to maintain an adequate weight).				
66. Is good value for money.				
67. Makes me feel good (mood).				
68. Is produced in Mexico.				
69. Does not contribute to CO^2 emissions.				
70. Is low in carbohydrates.				

**Item**	**Importance**			
All items begin with the following statement: *"It is important to me that the food I eat on a typical day..."*	Not at all	A little	Moderately	Very
71. Keeps me healthy.				
72. Is practical (easy preparation and consumption).				
73. Is from a commercial brand known to me.				
74. Contains no additives (added ingredients, sweeteners, colorants, preservatives, whether natural or not).				
75. Is not of animal origin.				

Items arranged according to the dimension they belong to:

**Dimension**	**Items (by number)**
Health and natural content (27 items).	56, 71, 57, 46, 65, 28, 58, 60, 15, 4, 74, 31, 36, 40, 55, 22, 13, 41, 63, 39, 70, 26, 21, 61, 66, 6, 48.
Environmental and wildlife awareness (12 items).	38, 19, 29, 44, 69, 49, 51, 75, 33, 64, 50, 10.
Image management (8 items).	73, 14, 3, 52, 23, 16, 1, 8.
Sensory appeal (6 items).	25, 5, 30, 45, 59, 20.
Price (5 items).	18, 12, 24, 9, 11.
Convenience (6 items).	47, 72, 43, 37, 17, 53.
Mood (5 items).	62, 67, 54, 34, 32.
Food identity (6 items).	7, 42, 27, 68, 35, 2.

Supplement S2-2. Updated-Food Choice Questionnaire (U-FCQ) in Spanish.

Por favor, para cada ítem seleccione sólo la opción que represente, en mayor medida, el grado en el que está de acuerdo con la afirmación correspondiente: "Es importante para mí que los alimentos que consumo en un día común..." Utilizando las siguientes opciones: "Nada importante"; "Poco importante"; "Algo importante"; o "Muy importante".

**Ítem**	**Importancia**			
Todos los ítems inician con la siguiente afirmación: *"Es importante para mí que los alimentos que consumo en un día común..."*	Nada	Poco	Algo	Muy
1. Sean libres de gluten.				
2. Sean parte de la dieta tradicional mexicana.				
3. Sean producidos por empresas que reconozca de la televisión, redes sociales u otros medios.				
4. Sean bajos en azúcares.				
5. Tengan un sabor agradable.				
6. Tengan el menor tiempo de almacenamiento (lo más frescos posibles).				
7. Sean productos locales o regionales.				
8. Sean considerados buenos por otras personas.				
9. Me mantengan sin hambre por varias horas.				
10. No hayan sido transportados distancias largas.				
11. Puedan ser comprados cerca de donde vivo o trabajo.				
12. No sean costosos.				
13. Sean ricos en fibra.				
14. Tengan una publicidad atractiva.				
15. Me aporten las calorías y nutrimentos necesarios.				

**Ítem**	**Importancia**			
Todos los ítems inician con la siguiente afirmación: *"Es importante para mí que los alimentos que consumo en un día común..."*	Nada	Poco	Algo	Muy
16. Vayan acorde a mis creencias religiosas.				
17. Me sean familiares o conocidos.				
18. Sean baratos.				
19. Sean producidos sin que los animales hayan sufrido dolor.				
20. Sean lo que usualmente como.				
21. Sean libres de endulzantes sin calorías (ejemplo: stevia, etc.).				
22. Sean recomendados por profesionales de la salud.				
23. Se parezcan a lo que consumía cuando era niño(a).				
24. Estén en oferta.				
25. Tengan un olor agradable.				
26. Sean de fácil digestión.				
27. Sean de temporada (propios de la estación del año como ciertas frutas y verduras).				
28. Sean bajos en grasa.				
29. Reduzcan la contaminación de suelo y agua.				
30. Tengan una textura agradable.				
31. Sean ricos en proteínas (ejemplo: huevo, frijoles, carne).				
32. Me ayuden a sobrellevar la vida.				
33. No contengan grasa de origen animal.				
34. Me mantengan despierto(a) o alerta.				
35. Sean consumidos por la mayoría de los integrantes de mi hogar.				

**Ítem**	**Importancia**			
Todos los ítems inician con la siguiente afirmación: *"Es importante para mí que los alimentos que consumo en un día común..."*	Nada	Poco	Algo	Muy
36. Contengan el menor número de sellos de advertencia en el empaque (ejemplo: exceso de sodio, calorías, grasa saturada).				
37. Puedan ser preparados fácilmente.				
38. Sean producidos respetando los derechos de los animales.				
39. Tengan información en la etiqueta nutricional del empaque que sea fácil de leer.				
40. Se apeguen al Plato del Bien Comer.				
41. Sean bajos en sal.				
42. Puedan ser consumidos en compañía de otras personas.				
43. Puedan ser consumidos casi inmediatamente.				
44. No ocasionen daños al medio ambiente.				
45. Tengan un aspecto agradable.				
46. Sean ricos en vitaminas y minerales.				
47. Puedan ser cocinados rápidamente.				
48. No me provoquen malestares físicos (alergias, dolor de estómago, diarrea, etc.).				
49. Utilicen envases o empaques amigables con el medio ambiente.				
50. No estén enlatados.				
51. Sean orgánicos (libres de fertilizantes y pesticidas).				
52. Reflejen a los demás una imagen positiva acerca de mí.				

**Ítem**	**Importancia**			
Todos los ítems inician con la siguiente afirmación: *"Es importante para mí que los alimentos que consumo en un día común..."*	Nada	Poco	Algo	Muy
53. Estén disponibles en tiendas, supermercados, tianguis, mercados.				
54. Me ayuden a reducir el estrés.				
55. No contengan ingredientes artificiales (conservadores, colorantes, sustitutos/imitaciones de alimentos, etc.).				
56. Sean nutritivos.				
57. Contengan en su mayoría ingredientes naturales.				
58. Sean buenos para mi piel, dientes, cabello, uñas, etc.				
59. Me sean placenteros.				
60. Me ayuden a controlar mi peso.				
61. Sean preparados en casa.				
62. Me levanten el ánimo.				
63. Sean mínimamente procesados.				
64. Mencionen claramente el país de origen.				
65. Sean bajos en calorías (me permitan mantener un peso adecuado).				
66. Tengan una buena relación calidad-precio.				
67. Me hagan sentir bien (estado de ánimo).				
68. Sean producidos en México.				
69. No contribuyan a la emisión de CO^2.				
70. Sean bajos en carbohidratos.				
71. Me mantengan saludable.				
72. Sean prácticos (fácil preparación y consumo).				
73. Sean de una marca comercial conocida para mí.				

**Ítem**	**Importancia**			
Todos los ítems inician con la siguiente afirmación: *"Es importante para mí que los alimentos que consumo en un día común..."*	Nada	Poco	Algo	Muy
74. No contengan aditivos (ingredientes añadidos, endulzantes, colorantes, conservadores, independientemente si son naturales o no).				
75. No sean de origen animal.				

Ítems agrupados de acuerdo a la dimensión a la que pertenecen:

**Dimensión**	**Ítems (por número)**
Salud y contenido natural (27 ítems).	56, 71, 57, 46, 65, 28, 58, 60, 15, 4, 74, 31, 36, 40, 55, 22, 13, 41, 63, 39, 70, 26, 21, 61, 66, 6, 48.
Preocupación por el medio ambiente y vida animal (12 ítems).	38, 19, 29, 44, 69, 49, 51, 75, 33, 64, 50, 10.
Manejo de imagen (8 ítems).	73, 14, 3, 52, 23, 16, 1, 8.
Atractivo sensorial (6 ítems).	25, 5, 30, 45, 59, 20.
Precio (5 ítems).	18, 12, 24, 9, 11.
Conveniencia (6 ítems).	47, 72, 43, 37, 17, 53.
Emociones (5 ítems).	62, 67, 54, 34, 32.
Identidad alimentaria (6 ítems).	7, 42, 27, 68, 35, 2.
