# Supplementary material for: Updated-Food Choice Questionnaire: Cultural Adaptation and Validation in a Spanish-Speaking Population from Mexico
Source: Nutrients. 2024 Oct 31;16(21):3749. doi: 10.3390/nu16213749 (PMC11548158; doi:10.3390/nu16213749)
Supplement: Supplementary file 1 [file nutrients-16-03749-s001.zip › U-FCQ Supplementary Table S1.pdf]

**Supplementary Table S1.** First and second panel of Nutrition professionals' characteristics.

| Variables | First panel (n=8) | | Second panel (n=13) | |
|---|---|---|---|---|
| | **n** | **IQR or %** | **n** | **IQR or %** |
| Age* | 30.0 | 3.0 | 33.0 | (11.5) |
| Sex† | | | | |
| Male | 2 | 25.0 | 2 | 15.4 |
| Female | 6 | 75.0 | 11 | 84.6 |
| Academic degree† | | | | |
| Bachelor's degree | 5 | 62.5 | 5 | 38.5 |
| Master's degree | 3 | 37.5 | 3 | 23.0 |
| Doctoral degree | 0 | 0.0 | 5 | 38.5 |
| Type of university affiliation† | | | | |
| Doctoral student | 8 | 100.00 | 5 | 38.5 |
| Full-time professor | 0 | 0.0 | 4 | 30.8 |
| Course professor‡ | 0 | 0.0 | 4 | 30.8 |
| Years working at the University* | 0 | 0.0 | 2.0 | 6.0 |

* Data are presented as median and IQR= Interquartile range.

† Data are presented as frequency and %= percentage.

‡ Professors that receive a payment per hours of class.
