# Supplementary material for: Updated-Food Choice Questionnaire: Cultural Adaptation and Validation in a Spanish-Speaking Population from Mexico
Source: Nutrients. 2024 Oct 31;16(21):3749. doi: 10.3390/nu16213749 (PMC11548158; doi:10.3390/nu16213749)
Supplement: Supplementary file 1 [file nutrients-16-03749-s001.zip › U-FCQ Supplementary Table S2.pdf]

Supplementary Table S2. Items modified from original Food Choice Questionnaire (11 out of 36), after face validation.

Original item (Steptoe, 1995)*	Nutrition professionals' assessment (clarity, relevance, representativeness and specificity)	Nutrition professionals' comments or suggestions	Resulted modification in Spanish	Modification in English (suggested translation)
Contains a lot of vitamins and minerals.	Not clear (n= 2)<br>Not specific (n= 1)	The term "a lot" is ambiguous.	Sean ricos en vitaminas y minerales.	Is rich in vitamins and minerals.
Helps me cope with stress.	Not clear (n= 1)	More than "cope" food helps to reduce stress.	Me ayuden a reducir el estrés.	Helps me reduce stress.
Is high in fiber and roughage.	Not clear (n= 2)	The term "high" is ambiguous.	Sean ricos en fibra.	Is rich in fiber.
Contains natural ingredients.	Not clear (n= 2)<br>Not specific (n= 2)	Clarify that it refers to the fact that they include mostly natural ingredients.	Contengan ingredientes naturales (en su mayoría).	Contains mostly natural ingredients.
Contains no artificial ingredients.	Not clear (n= 2)<br>Not specific (n= 1)	Specify what artificial ingredients are.	No contengan ingredientes artificiales (conservadores, colorantes, sustitutos/imitaciones de alimentos, etc.).	Contains no artificial ingredients (preservatives, colorings, food substitutes/imitations, etc.).
Contains no additives.	Not clear (n= 2)<br>Not specific (n= 2)<br>Not relevant (n= 1)	Explain the term "additives".	No contengan aditivos (ingredientes añadidos, endulzantes, colorantes, conservadores, independientemente si son naturales o no).	Contains no additives (added ingredients, sweeteners, colorants, preservatives, whether natural or not).
Is low in calories.	Not clear (n= 2)	Low in calories for what?	Sean bajos en calorías (me permitan mantener un peso adecuado).	Is low in calories (allows me to maintain an adequate weight).

Original item (Steptoe, 1995)*	Nutrition professionals' assessment (clarity, relevance, representativeness and specificity)	Nutrition professionals' comments or suggestions	Resulted modification in Spanish	Modification in English (suggested translation)
Makes me feel good.	Not specific (n= 1)	Specify that it refers to the emotional state.	Me hagan sentir bien (estado de ánimo).	Makes me feel good (mood).
Is easy to prepare	Not representative (n=1)	In Spanish, the term "preparation" does not take into account food cooking.	Sean prácticos (preparación fácil y consumo casi inmediato).	Is practical (easy preparation and almost immediate consumption).
Can be cooked very simply	Not specific (n= 1)<br>Not relevant (n= 5)<br>Not representative (n= 2)	Is similar to the item "Is easy to prepare"	Puedan ser cocinados fácilmente.	Can be easily cooked.
Takes no time to prepare	Not specific (n= 5)	Is similar to the item "Is easy to prepare	Puedan ser cocinados rápidamente.	Can be cooked quickly.

* Only modified items are presented, the rest (25 items) remained the same as in the original version. In English all items begin with the following statement: "It is important to me that the food I eat on a typical day…", whereas in Spanish, the following initial statement is used: "Es importante para mí que los alimentos que consumo en un día común...":
