# Supplementary material for: Updated-Food Choice Questionnaire: Cultural Adaptation and Validation in a Spanish-Speaking Population from Mexico
Source: Nutrients. 2024 Oct 31;16(21):3749. doi: 10.3390/nu16213749 (PMC11548158; doi:10.3390/nu16213749)
Supplement: Supplementary file 1 [file nutrients-16-03749-s001.zip › U-FCQ Supplementary Table S3.pdf]

Supplementary Table S3. Nutrition professionals' qualitative assessment of items that obtained a low Content Validity Ratio value (CVR <0.54).

Item (dimension)	Number of Nutrition professionals disagreeing (n=13)	Motive	Modification in Spanish (as validated)	Modification in English (suggested translation)
Gives people a good impression. (Image/opinion management)	6	Lack of clarity (n= 5)<br>Not relevant (n= 1)	Sean considerados buenos por otras personas.	"Is considered good by other people".
Does not cause me physical discomfort. (Health)	4	Lack of clarity (n= 4)	No me provoquen malestares físicos (alergias, dolor de estómago, diarrea, etc.).	"Does not cause me physical discomfort (allergies, stomach ache, diarrhea, etc.)".
Has an attractive advertising. (Image/opinion management)	5	Lack of clarity (n= 4)<br>Not relevant (n= 1)	Tengan una publicidad atractiva en su empaque.	"Has eye-catching advertising on their packaging".
Comes from countries I approve politically. (Ethical concerns)	6	Lack of clarity (n= 2)<br>Not relevant (n= 4)	Eliminated.	
Is low in carbohydrates. (Natural content)	4	Lack of clarity (n= 4)	Sean bajos en carbohidratos.	"Is low in carbohydrates". *
Is produced without human exploitation. (Ethical concerns)	6	Difficult to assess (n= 6)	Eliminated.	
Helps me cope with life. (Mood)	6	Lack of clarity (n= 6)	Me ayuden a sobrellevar la vida.	"Helps me get through life".
Has a long shelf life. (Convenience)	5	Lack of clarity (n= 5)	Tengan una vida de anaquel larga (no caduquen rápido).	"Has a long shelf life (does not expire quickly)".
Has not been transported excessive distances. (Sustainability)	5	Lack of clarity (n= 5)<br>Not relevant (n= 1)	No hayan sido transportados distancias largas.	"Has not been transported long distances".

* In its English translation, the item is unchanged.
