# Supplementary material for: Updated-Food Choice Questionnaire: Cultural Adaptation and Validation in a Spanish-Speaking Population from Mexico
Source: Nutrients. 2024 Oct 31;16(21):3749. doi: 10.3390/nu16213749 (PMC11548158; doi:10.3390/nu16213749)
Supplement: Supplementary file 1 [file nutrients-16-03749-s001.zip › U-FCQ Supplementary Table S4.pdf]

Supplementary Table S4. Discriminant validity analysis according to the Fornell and Larcker criterion (AVE-SV approach) [1].

Dimensions of the U-FCQ	M	HNC	SA	C	P	Fi	EWA	Im
Mood (M)	**0.30**							
Health and natural content (HNC)	0.44 (0.66)*	**0.41**						
Sensory appeal (SA)	0.32 (0.57)	0.28 (0.53)	**0.32**					
Convenience (C)	0.30 (0.55)	0.25 (0.50)	0.30 (0.55)	**0.35**				
Price (P)	0.19 (0.44)	0.12 (0.35)	0.16 (0.40)	0.27 (0.52)	**0.41**			
Food identity (FI)	0.29 (0.54)	0.40 (0.64)	0.24 (0.49)	0.23 (0.48)	0.17 (0.41)	**0.20**		
Environmental and wildlife awareness (EWA)	0.29 (0.54)	0.53 (0.73)	0.16 (0.40)	0.16 (0.40)	0.06 (0.26)	0.38 (0.62)	**0.39**	
Image management (Im)	0.15 (0.39)	0.18 (0.42)	0.09 (0.30)	0.12 (0.35)	0.10 (0.32)	0.28 (0.53)	0.24 (0.49)	**0.30**

Data are presented as shared variance (correlation coefficients) between factors.
The average variance extracted for each factor is presented in bold letters.
Abbreviations: AVE, average variance extracted; SV, shared variance; U-FCQ, Updated- Food Choice Questionnaire.
* Values for correlation coefficients ≥0.85 were considered as collinearity between factors [2].

References

1. Hilkenmeier, F.; Bohndick, C.; Bohndick, T.; Hilkenmeier, J. Assessing Distinctiveness in Multidimensional Instruments Without Access to Raw Data – A Manifest Fornell-Larcker Criterion. Front Psychol 2020, 11, doi:10.3389/fpsyg.2020.00223.
2. Rönkkö, M.; Cho, E. An Updated Guideline for Assessing Discriminant Validity. Organ Res Methods 2022, 25, 6–47, doi:10.1177/1094428120968614.
